# Modeling *TGF-β* in Early Stages of Cancer Tissue Dynamics

**DOI:** 10.1371/journal.pone.0088533

**Published:** 2014-02-20

**Authors:** Gianluca Ascolani, Pietro Liò

**Affiliations:** Computer Laboratory, Cambridge University, Cambridge, United Kingdom; Cedars Sinai Medical Center, United States of America

## Abstract

Recent works have highlighted a double role for the Transforming Growth Factor 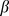 (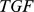-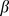): it inhibits cancer in healthy cells and potentiates tumor progression during late stage of tumorigenicity, respectively; therefore it has been termed the “Jekyll and Hyde” of cancer or, alternatively, an “excellent servant but a bad master”. It remains unclear how this molecule could have the two opposite behaviours. In this work, we propose a 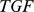-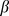 multi scale mathematical model at molecular, cellular and tissue scales. The multi scalar behaviours of the 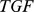-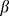 are described by three coupled models built up together which can approximatively be related to distinct microscopic, mesoscopic, and macroscopic scales, respectively. We first model the dynamics of 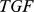-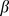 at the single-cell level by taking into account the intracellular and extracellular balance and the autocrine and paracrine behaviour of 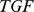-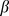. Then we use the average estimates of the 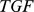-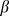 from the first model to understand its dynamics in a model of duct breast tissue. Although the cellular model and the tissue model describe phenomena at different time scales, their cumulative dynamics explain the changes in the role of 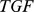-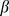 in the progression from healthy to pre-tumoral to cancer. We estimate various parameters by using available gene expression datasets. Despite the fact that our model does not describe an explicit tissue geometry, it provides quantitative inference on the stage and progression of breast cancer tissue invasion that could be compared with epidemiological data in literature. Finally in the last model, we investigated the invasion of breast cancer cells in the bone niches and the subsequent disregulation of bone remodeling processes. The bone model provides an effective description of the bone dynamics in healthy and early stages cancer conditions and offers an evolutionary ecological perspective of the dynamics of the competition between cancer and healthy cells.

## Introduction

A full systemic understanding of cancer process will benefit from investigating cell-tissue interaction. We can observe what happens at more or less all scales, from the disease at the whole organism down to the molecular level of cancer, and we have good amount of experimental data on all levels of biological organization. However, putting things together in order to obtain real understanding is much more difficult and much less developed. A way to build up multi scale models is by using proteins that are: 1) mutational drivers, meaning the mutation of one of the related genes causes the change of the phenotype, 2) able to interact with proteins which have intracellular and extracellular effects; hence, involving multi-cellular phenomena. Here, we start with the consideration that tissue modeling is the missing link between basic research and clinical practice, and we aim at using a modeling approach to bridge the cell to tissue scale in health and disease (cancer) dynamics. A key player of this multi scale process is 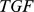-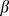 family of cytokines that control numerous cellular responses, including proliferation, differentiation, apoptosis and migration. 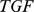-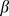 is always produced as an inactive cytokine that cannot bind to its receptor and function unless the latent complex is somehow activated. This regulation provides a complex control of 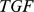-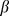 function, thereby ensuring that its potent effects are produced in appropriate locations and times. 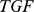-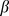 interacts with cytoskeleton, epithelial cadherin (E-cad) and integrins producing a multi scale mechanobiological effects on tissue [1]. Cancer is a multi scale, multifactorial and multi step process [2], [3]. The cancer cells undergo a cascade of mutations, some of them changing the phenotype, to obtain the ability to metastasise, and are constantly exposed to signals that induce apoptosis. Acquisition of antiapoptotic properties by cancer cells is important for metastasis, and recent studies suggest that 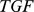-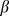 promotes the survival of certain types of cancer cells [4], [5]. 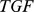-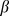 both inhibits and facilitates tumor progression during early and late stage of tumorigenicity, respectively. However, it still remains veiled how 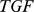-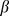 plays both contrasting roles [6]–[8]. Therapies based on 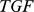-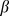 seem promising [9]. Tumorigenesis is in many respects a process of disregulated cellular evolution that drives malignant cells to acquire several phenotypic hallmarks of cancer, including the ability of growing autonomously, disregarding cytostatic signals, ignoring apoptotic signals, stimulating angiogenesis, invading, metastasising and becoming immortal. In the next section, we introduce the role of 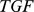-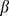 in breast cancer.

### The Ductal Lobular Unit and Breast Cancer

**Figure 1:**
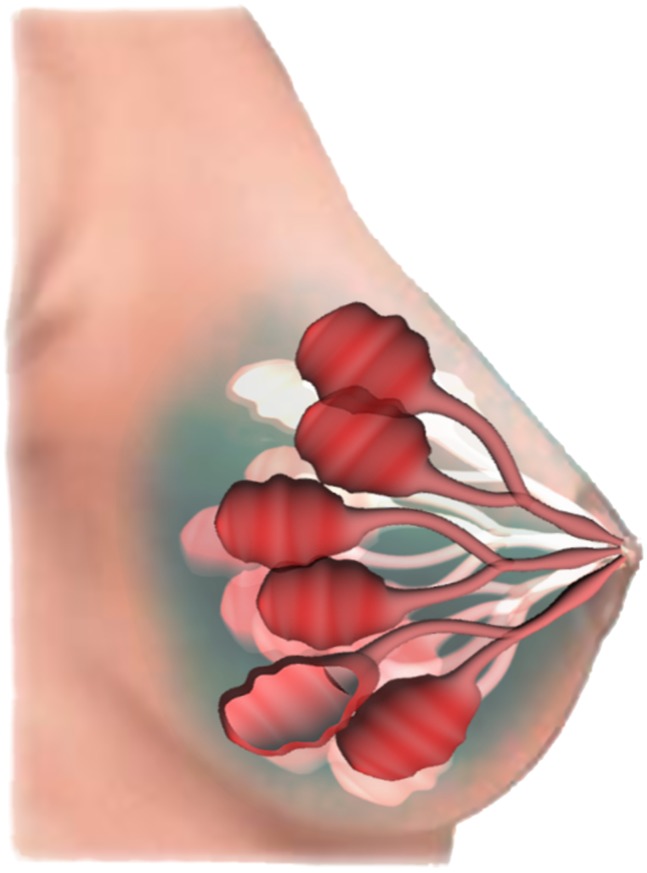
3D representation of the mammary duct. The mammary duct is formed by epithelial cells. Normal ductal cells (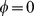) are regularly arranged on a single 2-dimensional layer. Tissue irregularities appear in presence of benign ductal cells, but cells still lie on a surface. When epithelial cells develop an aggressive phenotype (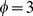), they begin to stratify.

The terminal ductal lobular unit is the basic functional and histopathological unit of the breast, and it has been identified as the site of origin of the most common breast malignancy. The ductal carcinoma corresponds to a specific stage of cancer development of the mammary parenchyma, Figure (1). Recent works showed that 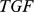-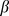 is abundantly expressed by highly metastatic breast cancer cells and promotes their survival. In particular, 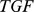-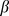 autocrine signaling, in certain breast cancers, promotes cell survival via inhibition of apoptotic signaling [10]. Major determinants of the “tissue identity” are the cadherins and integrins which are adhesion molecules regulating cell-cell and cell-matrix interactions. Cells containing a specific cadherin subtype tend to cluster together to the exclusion of other types, both in cell culture and during development. In vitro and in vivo studies have demonstrated the existence of crosstalk between integrins and cadherins in cell adhesion and motility.

Integrins play a key role in activating the latent complex of 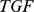-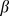. The binding of integrins to the latent complex induces a conformational change that produces active 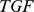-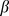 that binds to its receptor. Evidence now points to a crucial role for cell contraction in the activation of 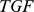-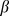 via few types of integrins. An intact actin cytoskeleton is required for 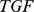-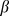 activation by these integrins; activation is greatly reduced by treatment of cells with the actin polymerization inhibitor cytochalasin. Additionally, treatment of cells with inhibitors of cell contraction, greatly reduces integrin-mediated 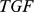-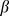 activation, whereas agents that stimulate cell contraction, such as thrombin, angiotensin-II and endothelin-1 enhance 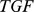-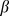 activation by integrins [1].

In breast cancer, the expression of E-cad is a hallmark of a well differentiated epithelium that functions to maintain cell-cell junctions, thereby inhibiting aberrant cell proliferation and migration. The loss of E-cad function via genetic inactivation by 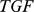-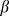 or via epigenetic silencing is a common characteristic of systemically invasive carcinomas. Down-regulated E-cad expression is required for the outgrowth of breast cancer cells. Breast cancer cells show a typical pattern of dissemination by 1) down-regulating E-cad expression or activity; 2) separating cell-cell junctions; 3) invading the surrounding tissues; and 4) intravasating the vasculature or lymphatic system [11]. Recent works show that down-regulated E-cad expression induced by 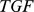-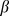 was sufficient to prevent mammary epithelial cell differentiation and instead, produced dense and more spherical cultures that underwent metastatic outgrowth [12] [13].

Here, we model the molecular and cellular mechanisms that underlie the 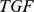-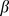 capacity in suppressing tumor development in normal cells, and conversely, to facilitate cancer progression and increase number of malignant cells. Our multi scale model focuses on the 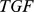-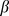 activation requirement, its autocrine properties of 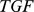-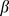, its role in promoting cell contraction and being activated by cell contraction [6]. First we model the single cell and then the cell population by investigating the autocrine and paracrine of 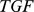-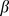 in a generic epithelial tissue. We investigate the relation between the concentration of 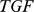-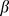 and its receptors with respect to the stage of the cancer. In this perspective, we model the early invasion of few malignant cells in a healthy tissue and the different role of autocrine and paracrine 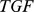-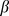 secretion. The autocrine and paracrine behaviour are explored on the light of the evolutionary ecology principle of malignant and healthy cell competition. It is known that the bone tissue is the preferred niche of breast cancer colonization; we present a model of the role of 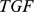-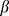 in bone invasion and alteration of bone tissue remodeling dynamics.

In summary, here, we introduce a set of scale specific mathematical models that render the multi scale behaviours of 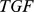-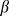 allowing us to describe the early breast cancer development and the initial condition of the metastasisation process by using a level of description familiar to biologists in order to encourage experimentation and hypothesis testing.

## Results

The development and spread of cancer, although caused by driver mutations producing variations in gene expression and signaling disfunctions, involves cytoskeleton biomechanical changes that modulate cell dynamics at the tissue level. The development of a tissue mathematical model requires considering the 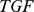-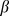 autocrine and paracrine behaviour of the cells. Therefore, we focused on the interface between intracellular and extracellular compartments. Given the different time dynamics of the reactions at the intracellular pathways level and the cell dynamics at tissue levels, we prefer to build distinct models, coupled by time averages of the fastest dynamics. Following the model developed by Laise et al. [14], we have focused on autocriny and paracriny behaviours of the 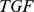-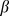. Next, we have considered a tissue model to describe the effects of the 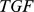-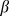 on cellular populations characterized by different driver mutations. Finally, we consider the bone niche model which allows us to describe the effects of the tumoral cells on the BMU (Basic Multicellular Unit) remodeling cycle. Each of these models describes different aspects of the 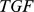-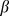 at a particular scale and they are loosely coupled by using averaged quantities of 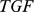-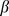 in such a way to mimic the interactions between different scales; This allows us to consider each model as a “sub-model” which is part of a more comprehensive multi scale model. From here on, when we refer to the multi scale model, we will use the single term model, and we will specify otherwise when referring to one of the scale specific model. Describing the dynamic development of tumors requires the knowledge of numerous degrees of freedom, which often are not experimentally available, and a complex model able to correctly analyse all the data, retrieve the relevant information of the present state and simulate its future evolution. Here, we propose a model to explain the early stages of tumor and its evolution in bone tissue based on production and sensing of 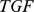-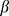 in both the paracrine and autocrine processes.

### The Intracellular Generation of 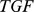-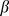 Fluxes

In this work, we follow the model of Laise et al. and [15], [16] that for the sake of completeness, we will re-propose and adapt into the structure and the aims of a multi dimensional model that embrace both the intracellular/cell and cell/tissue interfaces. They considered a simple biochemical model which explicitly describe the key features of the Smad pathway. The first three equations of the model, see system of equations (1), accounts for the interaction between 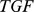-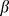 (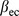) and the inactive membrane receptor (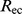). Following a successful encounter, the receptor turns into its active form here denoted 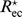. In reality, different isoforms of 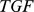-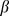 and of its receptor exist. In [14], the authors simplify the model setting by considering just one receptor type, which can operate in its active (bound to 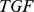-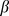) or inactive configuration. The activated receptor is able to phosphorylate the 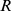-Smad proteins (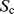) in the cytoplasm, resulting in the formation of a new species, the phosphorylated Smad protein, here labelled 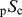. Once phosphorylated, the Smad proteins head towards the nucleus. The translocation of the Smad proteins into the nucleus (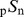) is necessary to activate the transcriptional activity. This is a complex process, possibly organized in cascade regulatory cycles. Note that cytoplasmatic 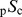 are modified into nuclear 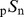 with a rate specified by the control parameter 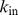. The presence of phosphorylated Smad in the nucleus is in turn associated to an increase of the 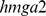 gene expression [17]. The nucleus is also enriched by phosphates (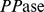) targeting phosphorylated Smad [16]. The dephosphorylation of the nuclear Smad proteins 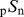 results in non-phosphorylated nuclear Smad elements 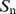. The model is composed of eight variables, respectively 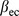, 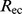, 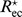, 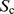, 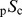, 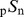, 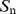 and 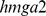. The associated concentrations obey to the following set of ordinary differential equations:
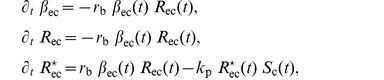
(1)




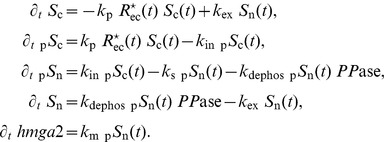
where the notation 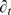 represents the partial derivative in respect to the dependent variable 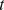 indicating the variation in time of the following quantity on the right. In the last equation of the system (1), 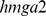 measures the mRNA amount and as such assumes arbitrary units. All the other concentrations are instead expressed in nmol. Notice that the concentration of 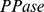 remains unchanged all along the dynamical evolution. Asymptotically, the system admits a family of stable fixed points. From the experimental results in [16], eventually the 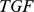-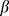 amount as well as the activated receptors amount go to zero, and consequently, there is only one fixed point that satisfied such conditions. Analogously, according to the model specification, we have 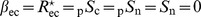. The quantities 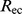, 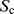 and 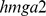 converge instead to stationary solutions, function of the initial condition and of the kinetic parameters involved. The 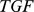-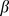 pathway is in particular reduced to a limited set of meaningful chemical reactions that are presumably implicated in the transmission of the signal from the cell surface, as triggered by 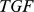-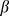, to the cell nucleus. The analysis conducted by the authors in [14] is in particular aimed at inspecting the out-of-equilibrium dynamics of the system, as driven by the externally imposed 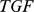-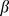. The model proposed by Laise et al. for the epithelial-mesenchymal transition predicts the concentration of mRNA associated to gene 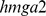, properly describes the results of the in-vitro experiments set up by [16] and reproduces the right unperturbed steady state characterized by specific concentrations of cytoplasmatic Smad proteins 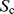 and unbound receptors 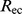, which have been carefully evaluated by Schmierer and collaborators.

To address the problem of building a model which takes into consideration the effects of the 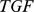-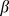 pathway signaling and tumor regulation at different scales, we adopt this intracellular model as a starting point. In our multi scale approach, we introduce two main re-adaptation of the previous intracellular model. First, we have done a model order reduction regarding the Smad and the 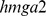 concentrations by imposing 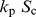 constant in time, which allows us to simplify the intracellular processes disregarding the effect of the Smad signaling cascade reactions, see equations (1) in the dashed line box, in order to focus on the 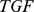-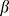 pathway, see equations (1) in the continuous line box. The justification for such approximation, as explained in [14], is due to the large amount of 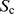 in comparison to 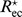 and to the re-integration of 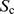 which permits us to consider the variation of the Smad in the cytoplasm negligible. Second, for a description of processes that occur not only at different spatial scales, but also at different time scales, we need to introduce source terms (and sink terms when necessary) for the synthesis of both the 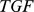-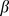 and its receptors in order to move the fixed point in such a way the remaining quantities, at intracellular scale, are all different from zero. This is much more reasonable for long and continuously ongoing processes; while positive quantities, like the concentrations, approaching zero implies the processes will come to a standstill irremediably affecting the system at all scales.

### The Cellular Model

The cellular model is the first of the three models where we discuss the intracellular/cell interface and take into consideration the dynamic effects of the 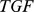-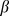 in a small patch of cells. With this model, we address the problem of production and internalization of 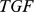-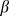 along with the binding of autocrine or paracrine 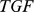-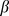 to the receptors on the membranes without considering the detailed spatial disposition of cells. Even though the autocrine and paracrine signaling are completely distinct forms of exchanging chemicals, it is impossible to distinguish between the two when they occur at the same time. It is true that a cell can sense the local spatial inhomogeneity of chemicals and the heterogeneity of and positions of other cells it is in contact with; hence, the cell can regulate itself to secrete the chemical compounds along preferred directions in such a way the chemicals will most probably follow an autocrine pathway or a paracrine one. Nevertheless, these cellular behaviours and this level of detail are unknown and unavailable for the 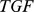-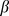 signaling.

On the other hand, it is important to stress that, if all the cells have similar behaviours in respect to the 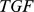-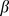 secretion/absorption, and they are homogeneously distributed, then the average paracrine signaling to and from other cells cancel out one another. In the case proposed here, the paracrine signaling inward and toward the neighbour cells do not cancel out because cells with different phenotypes do not behave in the same way. To overcome such difficulty, we propose to subdivide the space where cells are placed by adopting a “cell-centric” point of view. Therefore, for each cell, we consider a spheric volume containing and centred on the cell itself. The surface of the cell membrane divides this volume between the intracellular part and the external part. The latter is divided in the extracellular region, which is the closest neighbourhood just around the surface of the cell membrane where the 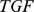-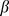 produced by the cell is released and can bind with the receptors of the specific cell at the centre of the volume, and the diffusive region representing the farthest part of the spherical volume from the cell where the 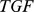-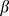 cannot reach the receptors on the cell surface of any cell. Between two nearest cells, the farthest parts of the respective diffusive regions are the intangible frontiers where the two “cell-centric” spatial subdivisions join together. Furthermore, we extend the previous definition by considering the diffusive region of each cell as the place where all surround “cell-centric” spaces join together; therefore, the diffusive region is the common area between a cell and all its nearest neighbour cells. The 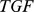-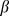 entering in the diffusive region loses the possibility to bind with cells and also loses any dependency on the cell from whom it has been produced. In other words, the 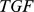-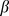 produced by the cells, which does not bind autocrinely, flows before into the diffusive region and then flows back toward all the cells in the neighbourhood indistinctly, so that it can bind in a paracrine way.

The portion of volume occupied by the union of all the external parts depends on the volume of the cells and the distances between their membranes. This intercellular space is filled with the extracellular matrix (ECM), a fibrous mesh which gives it the peculiar behaviours of the porous media. The ECM is responsible for the reduced diffusivity of the active 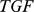-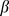. As a consequence, the 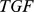-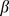 in the diffusive region cannot easily diffuse toward the cells, and the 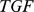-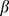 in the proximity of the cells is more easily conveyed toward the receptors on the cell surface. The prolongated time of diffusion is also responsible for the dispersion of 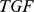-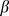. The obstructions in the porous media introduce non-linearity in the diffusion equation. Excessive non-porosity can forbid the diffusion of molecules through the media, or it will oblige molecules to follow highly crooked paths. To avoid excessive complications in the diffusion of 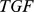-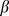 due to the complex geometry produced by the disposition of the fibrous mesh, we can take into consideration the averaged conformational characteristics of the ECM in the diffusive region by using an effective diffusion coefficient which can be experimentally measured. Furthermore, the compartmental description of the ECM as a region with impaired diffusivity also reproduces the function of storing growing factors.

Using the first Fick’s law [18], the flux of molecules of 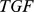-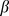 crossing a unitary orthogonal surface is given by 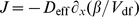 where the effective diffusion coefficient 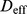 includes the porosity of the ECM, and the density of 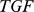-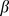 is expressed as the quantity of molecules over the diffusive region volume 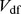. Discretizing the first Fick’s law and multiplying both sides of the equation by the orthogonal surface 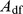 crossed by the 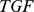-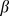 during the diffusion between the extracellular region and the diffusive region, we get 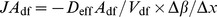 where 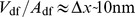 is the distance between two adherent cells whose neighbouring membranes are bridged by E-cad. The quantity of molecules escaping through the orthogonal surface per unit of time can also be expressed in terms of the paracrine rate 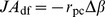 and hence, the 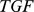-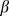 paracrine rate is equal to 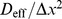.

We want to remark that the introduction of the diffusive region makes it possible to mimic the paracriny between cells, but at the same time, we completely disregard the relative disposition between cells. Such approximation is not entirely appropriate to describe the exchange of 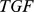-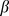 between two specific cells. However, it is justifiable when describing an averaged effective exchange of 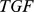-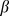 between different groups/types of cells.

After the 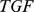-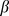 binds with the receptors, a series of reactions involving the Smad proteins follow their internalization. For the sake of simplicity, we do a model order reduction on the intracellular model proposed in [14] by considering the non-phosphorylated Smad proteins in the cytoplasm constant in time, see last equation in the continuous line box and first equation in the dashed line box of the system of equations (1).

Due to the importance of 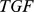-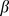 for different aspects of the cellular life cycle, degradation of 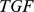-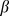 in the intracellular compartment can not be neglected especially in healthy cells where the over-accumulation of 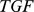-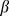 can produce a large disregulation. Therefore, we consider that healthy cells ubiquitinate part of its 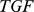-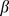, while mutated cells do not perform such activity. The set of partial differential equations for the cellular model are:
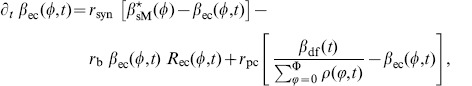
(2)




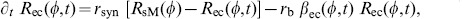
(3)




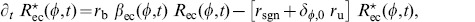
(4)




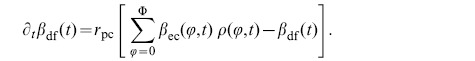
(5)


The system of equations (2–5) defines the evolution in time of the 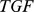-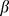 produced (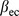), its receptor on the cell membrane (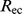), the internalised 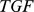-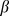 (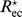) and the 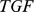-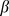 in the diffusive region (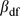), see Table (1). While the 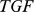-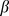 in the diffusive region represents the total amount of 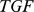-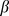 that all the cells in the nearest neighbourhood are paracrinely exchanging, the other variables are intended as averages all over the sub-populations of cells with the same phenotype. Therefore, the quantities 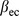, 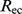 and 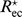 depend on the phenotype expressed by the index 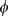 (and 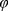). The region of interest within the tissue where the tumor begins to develop is also partitioned in sub-populations of cells, 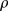, identified by the phenotype index which goes from 0 to a maximum value 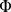. The phenotype index is associated with the cell aggressiveness and sensibility to the 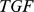-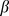 so that 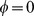 corresponds to the healthy cells and as 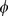 increases, the more cells are aggressive and need more 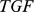-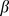 in order to respond to its signal.

**Table 1:** List of variables.

variable/unknown	symbol	initial value
extracellular 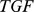 - 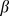	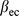	{2,2,2,2} [nmol]
receptors on the membrane	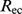	{2,2,2,2} [nmol]
internalized receptor-ligand	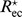	{1,1,1,1} [nmol]
autocrine 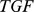 - 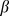	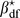	0.1 [nmol 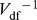 ]
number of nearest neighbor cells with phenotype 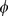	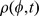	{6,0,0,0} 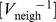
osteocytes	*Ocy*	900 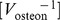
osteoclasts	*Oc*	0 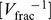
lining cells	*Lng*	0 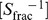
osteoblasts	*Ob*	0 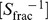
RANKL	*RANKL*	0 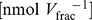
BMP	*BMP*	0 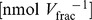
CSF	*CSF*	0 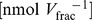
BMD	*BMD*	100 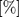

If the variable depends on the phenotype 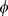, then we give a list of values sorted by increasing order of the phenotype. References and choices for the numerical values of the initial values are discussed in the data analysis section.

In equation (2), the first two terms describe the synthesis of 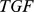-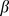 that is secreted in the external region where it is activated and the binding between the 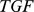-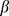 and its receptor. The third term takes into account the averaged values of 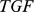-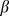 transferred by one cell with phenotype 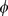 toward to the diffusive region and the mean 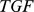-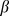 per cell received from the diffusive region. Similarly, equation (3) describes the synthesis of 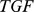-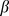 receptors which are displaced on the cell membrane and binds with the 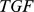-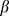 present in the extracellular region. The 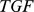-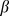 binds to its receptor on the cytoplasm membrane, and it is internalized. Inside the cell, the 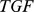-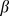 interacts with the Smad [14] at rate 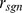, and to avoid an excessive abundance of this protein, ubiquitination occurs with rate 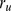, see equation (4). The operator 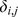 in equation (4) is a delta of Kronecker which takes value 1 when the indexes 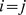 and zero when the two indexes are different. The variation of total 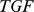-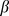 in the diffusive region is due to the incoming 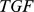-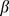 which each cell exchanges paracrinely and the out coming flux shared among all the nearest neighbour cells, first and second term in equation (5) respectively.

**Figure 2:**
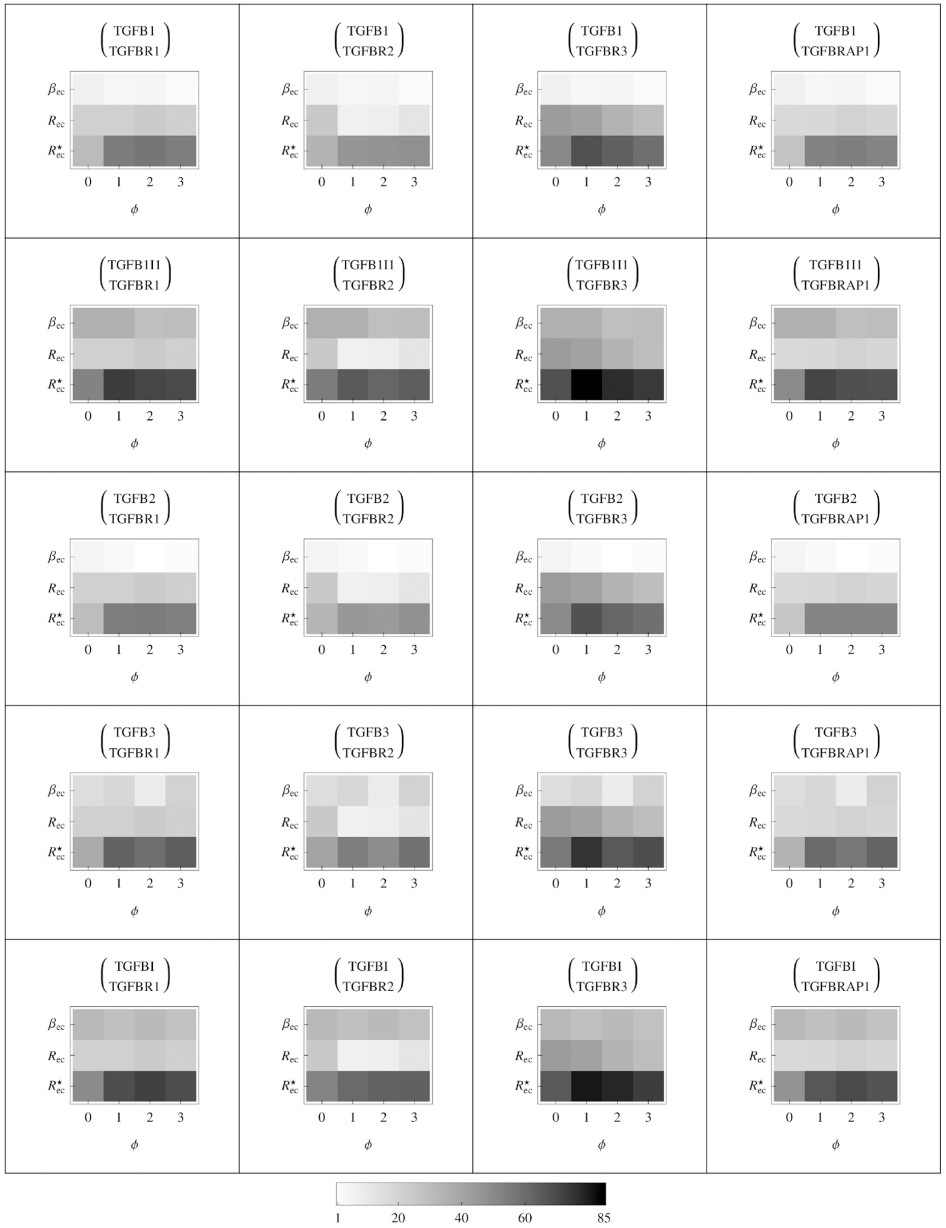
Table of 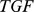-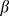 synthesized, 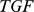-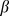 receptor synthesized and 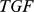-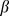 internalized for each phenotype 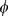. These values are obtained from the numerical solution of the cellular model equations (2–5) at time 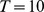 days. The various types of 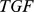-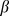 and receptors are expressed on top of each box. The tumor grades are sorted into increasing order of severity and aggressivity.

We have used the 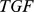-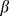 and 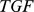-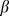 receptors gene expression data in the cellular model equations (2–5) to evaluate the respective synthesized quantities and the concentration of the active 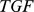-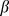 in the intracellular compartment for each sub-population with phenotype index in the range 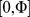. In Figure (2), we show the results for different 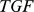-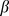 isoforms. Due to the disregulations of 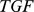-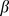 production in cells with driver mutations, we can observe that the concentration values of 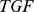-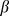 fluctuate between healthy, pre-tumoral and tumoral cellular phenotypes, and there is no clear trend which follows the severity of the tumor. On the other hand, some particular cases are characterized by a monotonic relationship between the populations’ phenotype and the concentration of the 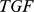-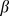 quantities, see the 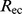 for the isoforms TGFB1I1/TGFBR3, both 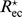 and 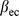 for the isoforms TGFB1/TGFBR2, the 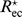 for the isoforms TGFBI/TGFBR2 and the 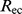 for the isoforms TGFB3/TGFBR3. These particular isoforms are useful to identify the tumor cell phenotype. For example, the expression for the TGFB1I1 receptors, which have an antitumorigenic effect, decreases with the increase of cellular aggressiveness.

It is important to stress that the dynamics of the 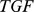-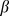 pathway of a sub-population with a specific phenotype described by the system equations (2–5) depends on the dynamics of the 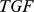-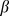 of the other cellular sub-populations in two different ways. The first one is the paracrine exchange of 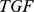-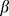 with rate 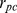 between the sub-populations expressed in equation (2) and equation (5). The second way the equations (2–5) are coupled between different phenotypes with one another is given by the average number of nearest neighbour cells 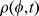. The former describes a cellular scale phenomenon, while the latter is a tissue scale phenomenon. Hence, if the number of cells of the different sub-populations is not constant, but changes dynamically, then it is necessary to supply the cellular model with a set of equations for the cellular evolution of the tissue (see next subsection for the tissue model).

### The Tissue Model

Mutations are responsible for the behavioural changes of cells that, from a healthy state in which they are capable of correctly sensing and responding to the surrounding signaling, enter to a mutated state, where the cells cannot self-regulate in response to the homeostatic signals. Mutations induced by external agents, or due to the occurrence of casual variations in the DNA’ s transcription while proliferating, can be easily accumulated during a cell life or in multiple progenies. Hence, the increasing in the population number and the survival of mutated cells can obstruct the tissue integrity and its functional activity well before the cells acquire a highly malignant phenotype. As previously stated, the 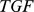-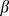 signaling has a pivotal role in maintaining homeostasis at the cellular scale and the functional integrity at the tissue scale. Indeed, the 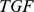-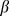 downregulates the cell proliferation, is responsible for the cell cohesion at high concentration, induces cell apoptosis [7]. Nevertheless, the anti-tumorigenic mechanisms provided by the 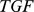-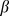, and their effectiveness, depend on the capability of the cells to properly sense its signal. DNA mutations (in particular, driver mutations [19]), on one side, can induce changes in cell phenotype which destabilize the correct cell functions. The weakening of a cell and loss of its stability are responsible for the increase of active 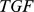-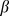. On the other side, mutations can produce a resistance of the cell response to the 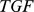-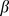 signaling. Cells with these driver mutations can also acquire the capability to produce a higher concentration of 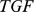-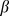 which is required to reach a different homeostatic level without incurring in apoptosis. Eventually, a cell can always undergo a mutation resulting in the failure of the anti-tumorigenicity of the 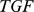-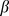 and in an inversion of its role, meaning the transformation of 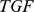-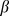 from Dr Jekyll to Mr Hyde occurs [8]. In the latter case, the 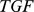-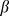 fails in downregulating cell proliferation and inducing cell apoptosis; while the excessive production of 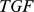-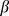 becomes dangerous for the surrounding cells which have not yet acquired sufficient resistance to the growing factor.

As previously said, cell mutations are random, and each mutation can induce apoptotic cell resistance to certain signals, or it can introduce cellular instability and put the cell to death. Nevertheless, this does not mean there is no relation between mutations, or that a mutated cell can accumulate mutations and return to its original state. Indeed, the cell behavioural changes induced by mutations can be associated to a stage. This cell stage indicating the results of mutations does not variate continuously, but, on the contrary, it is characterized by abrupt phenotype changes. Therefore, mutations make the cell change from one stage to another with given probabilities, and the presence of correlations or constraints between mutations oblige the cell each time to variate its stage going through a small subset of all the possible mutation states. Indeed, the mutation state of a cell monotonically increases during its life, producing a progressive change of its phenotype which from a normal stage goes through a series of pre-neoplastic steps to a neoplastic phenotypical stage.

On the other hand, not all the occurrences of a mutation imply a change of the cellular phenotype or a change in the production of and response to the 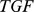-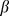. Furthermore, at each stage, a cell has a given probability in acquiring a complete resistance to the tumoral suppressor action of the 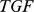-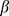 by switching to a phenotype where the 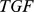-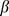 becomes a tumoral promoter. To address the differences between cell tumoral behaviours and cell response to 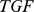-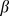, we introduce a discrete positive variable 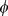 that represents the cell phenotype. The index 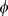 subdivides cells into groups which share the same phenotype without considering the specific mutations accumulated by each single cell; therefore, each cell in a group has the same sensibility to the signaling induced by the active 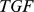-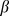 bound to the membrane’s receptors and activates the same amount of 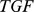-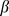 per unit of time. From a biological point of view, in our prospective, the values of 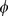 has some relativeness with the hallmarks of cancer [2], [3], and from a medical point of view, it may be related with the diagnostic stage of cancer.

All quantities and characteristics which refer to a particular cell stage, or mutated cell population, will depend on the phenotype 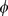. Therefore, normal cells have a phenotype 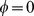, pre-neoplastic cells correspond to 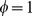, tumoral cells are indexed as 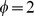, and cells with aggressive tumoral behaviours and strong resistance to 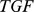-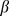 inhibiting signaling have phenotype 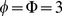, [20], [21]. A possibility of explaining the different responses to 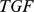-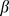 signaling by cells with different phenotypes is thinking that a normal healthy cell has 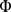 different mechanisms working in series to regulate the response/sensing of 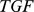-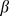. Therefore, at the beginning, the signal 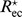 entering the cell is amplified to 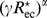 where 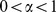 and 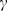 is a suitable scaling constant. Then, the amplified signal is sent to all the 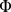 mechanisms and a binary information 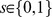, which indicates if there is an absence or presence of entering 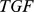-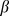 respectively, is sent to the first regulating mechanisms in the series. Each mechanism uses the entering signal to amplify the binary information 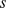 of a factor 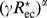. After all the 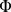 mechanisms are applied, the outcome 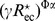 is compared with the amplified signal 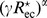 and their ratio is used as an upregulation of the apoptotic signaling and as a downregulation of the cell proliferation. For a normal cell (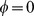), all the 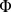 mechanisms regulating the response of the 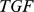-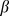 are functioning, while for the successive phenotype 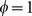, there is one mechanism which always fails to function. The failure of the mechanism results in no contribution to the amplification of the binary signal. As the phenotype 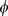 increases so does the number of failing mechanisms. When all the mechanisms which amplify the entering signal are non-functioning due to the severe mutations like in aggressive tumoral cells, the binary signal 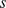 (non amplified) compared with the entering signal 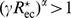 produces a reduction/dumping of the 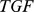-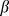 signaling. Consequently, the cells respond by increasing the proliferation and decreasing the apoptosis.

To describe the dynamic evolution of the cell populations for each phenotype 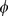, we propose a model based on the effectiveness of the cell response to the 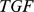-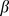 signaling. To easily describe the exchange of 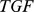-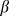 between cells and their phenotypical evolution, we focus our attention on a small region of healthy tissue in which cancer cells will form. Precisely, we consider a volume containing a cell, all its neighbour cells and part of the empty space inside the mammary duct, see Figure (1). Cells in a normal mammary duct are regularly arranged in such a way to form a tubular surface one cell thick. Even when cells start to present some pre-neoplastic phenotypical behaviours, they continue to lay on the mono layer duct surface, but with a less regular arrangement. Therefore, after having set the volume of the region of interest, we can consequently fix the average maximum number of cells, 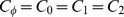, which can lay on the surface of the mammary duct. On the other hand, neoplastic epithelial cells in the mammary duct tend to form multi-layers; therefore, the volume capacity, 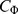, of this type of cells is bigger than in the other cases. The average number of cells 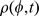 within the volume of interest is described by the following equation: 
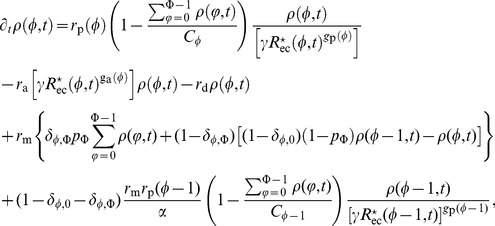
(6)with the additional condition that 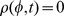 for 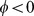 (the phenotypes 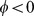 are set constantly equal to zero, because they do not have any biological meaning and they can be disregarded). The first term on the right hand side of equation (6) describes the proliferation limited by the volume capacity 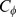, and downregulated by both the 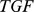-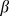 entering the cell, 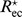, and the capability to respond to it, 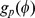. The second and third terms express the cellular death induced by the 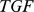-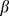 signaling, which also depends on the phenotype sensing exponent 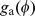, and by the cell instabilities induced by random mutations. The fourth term describes the changes of phenotypes (the increase of 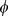) as a consequence of the mutations. The delta of Kronecker shows that normal cells can only develop anomalous behaviours, and aggressive cells (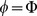) do not change their phenotype. Similarly, the fifth term describes the occurrence of important mutations and of phenotype changes during the cell proliferation. We introduce a upper limit 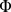 for the phenotype values because when cells accumulate to many mutations, they reach a stage of instability which are inconsistent with both the aggressiveness of the cell phenotype and the diagnostic stage of cancer. A similar upper limit used to label the tumoral stage of the cancer cells has been adopted in [22] as the limit in which cells are prevalently characterized by an apoptotic regime instead of those characteristic hallmarks associated to cancer development [2], [3].

**Figure 3:**
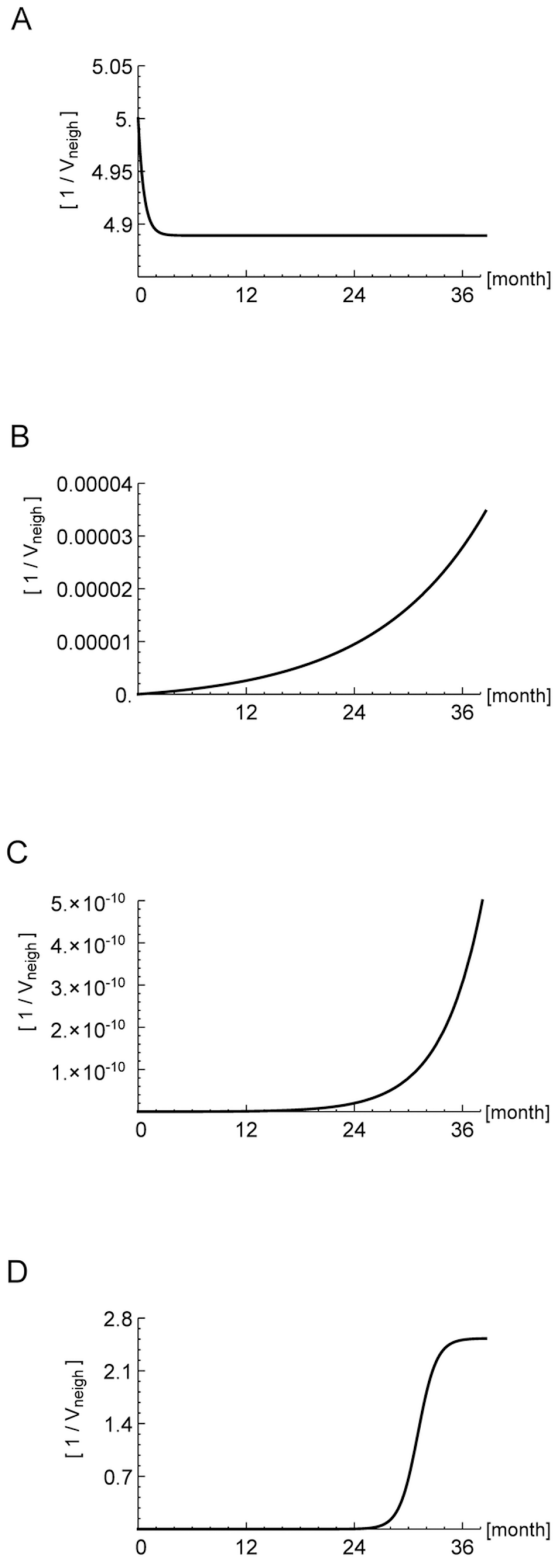
Evolution in time of the average sub-populations’ densities. Given a volume 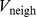 of a size equal to the average volume that, in a healthy mammary duct tissue, contains exactly a cell and all its nearest neighbour cells arranged on one layer, the densities for each sub-population in the volume 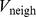 is shown. Starting from the top, A) the healthy cells, B) the pre-neoplastic cells, C) the tumoral cells and, D) the aggressive tumoral cells are graphed respectively. On the abscissa the time in months.

In Figure (3), we show the numerical solution of the tissue model coupled with the cellular model. The figure represents the evolution of the average nearest neighbour cell sub-populations’ densities inside the mammary duct. The occurrence of driver mutations inducing phenotype variations and the following disregulation of 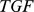-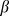 cell production results in an increase of cell populations with higher phenotype indexes to the detriment of the healthy cell population, Figure (3.A). In Figure (3.B) and Figure (3.D), we can see that pre-tumoral cells, having developed only a partial resistance to 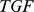-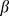 signaling, proliferate much slower than cells with aggressive tumoral behaviours. Nevertheless due to rare occurrences of driver mutations inducing phenotype changes, the cell population with phenotype 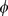 equal to 2 increases much slower than pre-tumoral cells, Figure (3.C). In Figure (3.D), the tumoral cell sub-population with phenotype 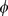 equal to 3 is characterized by a long initial part where they are almost zero followed by a high proliferation phase until the maximum cell capacity is reached. This shows that, even though 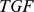-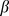 has lost its anti-tumorigenic role on aggressive cells by increasing their proliferation and driver mutations immediately changing the cell phenotype to the maximum value 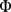 can occur at any moment, the delay with which aggressive tumoral cells form points out that they are prevalently originated by sub-populations which have already developed 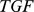-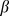 resistance more than by healthy cells. Hence this highlight the strong capability of 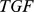-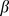 in slowing down the cancer development by acting on pre-tumoral cells. On the other hand the steep increase of aggressive cancer cells, after the first plateau-like phase, remarks the role of 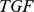-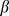 as cancer promoter on aggressive tumoral cells.

It is important to point out that both the cellular model and the tissue model describe the dynamic of averaged quantities. Also the coupling between the two is regulated by the average of the 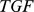-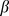 entering the epithelial cells and the average numbers of neighbour cells with a specific phenotype which compose the mammary duct tissue in the region of interest. The differences in the order of magnitude between the rates in the cellular model with respect to tissue model, see Table (2), allow us to overcome such difficulties; in fact, phenomena occurring at the cellular scale are much faster and relax to a steady state more rapidly than the events happening at the tissue scale. Hence the difference between the two time scales and the loosely coupling, due to averaged 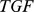-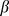 quantities, makes it possible to reduce the coupling between the extracellular and the tissue scales resulting in multi scale model consistent with the biological phenomena.

**Table 2:** List of parameters.

parameter	symbol	value
gene expression of the TGF-*β*	*β*_sM_( 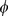 )	{–1.853, 1.724, 1.614, 1.669} [nmol]
gene expression of the receptors	R_sM_( 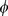 )	{–7.47, 6.713, 5.017, 4.128} [nmol]
rate of synthesis	*r*_syn_	133.333 [s^−1^]
Smad signaling rate	*r*_sgn_	0.003018 [s^−1^]
paracrine rate	*r*_pc_	10 [s^−1^]
binding rate	*r*_b_	0.0074 [nmol^−1^ s^−1^]
ubiquitination rate	*r_u_*	0.007 [s^−1^]
total number of phenotypes and malignant phenotype	Φ	3
proliferation rate	*r*_p_( 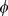 )	{–1. ×10^−6^, 1. ×10^−6^, 1. ×10^−6^, 1. ×10^−6^} [s^−1^]
apoptosis rate	*r*_a_	9. ×10^−8^ [s^−1^]
degradation rate	*r*_d_	1. ×10^−8^ [s^−1^]
mutation rate	*r*_m_	1. ×10^−14^ [s^−1^]
proliferation exponent	*g*_p_( 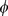 )	{–0.12, 0.06, 0.,−0.06}
apoptosis exponent	*g*_a_( 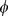 )	{–0.12, 0.06, 0.,−0.06}
probability of malignant mutation	*p*_Φ_	5. ×10^−7^
cell capacities	C_ø_	{–6, 6, 6, 8} [*V*_neigh_^−1^]
dimensional constant	α	1 [s^−1^]
dimensional constant	γ	1 [nmol^−1^]
Osteocytes formation rate	f*_Ocy_*	0.00032 [*S*_frac_ *V*_osteon_^−1^ day^−1^]
Osteocytes apoptosis rate aOcy	*a_Ocy_*	30 [*V*_osteon_^−1^ day^−1^]
Osteoclasts recruitment rate	*r_Oc_*	0.8 [*V*_frac_ nmol^−2^ day^−1^]
Osteoclasts apoptosis rate	*a_Oc_*	0.0625 [day^−1^]
Lining cells proliferation rate	*p_Lng_*	50 [*µ*m nmol^−1^ day^−1^]
Lining cells maturation rate	*m_Lng_*	50 [*µ*m nmol^−1^ day^−1^]
Lining cells apoptosis rate	*a_Lng_*	0.1525 [day^−1^]
Osteoblasts apoptosis rate	*a_Ob_*	0.1525 [day^−1^]
RANKL production (Osteocytes)	*p_RKL_*	300 [nmol *V*_osteon_ *V*_frac_^−1^ day^−1^]
RANKL production (osteoblasts)	*r_RKL_*	2.5 [nmol *S*_frac_ *V*_frac_^−1^ day^−1^]
RANKL degradation rate	*d_RKL_*	8.6643 [day^−1^]
BMP production rate	*p_BMP_*	1 [nmol *V*_osteon_ *V*_frac_^−1^ day^−1^]
BMP degradation rate	*d_BMP_*	8.6643 [day^−1^]
CSF production rate	*p_CSF_*	0.1 [nmol *V*_osteon_ *V*_frac_^−1^ day^−1^]
CSF degradation rate	*d_CSF_*	69.3147 [day^−1^]
Bone resorption rate	*r_bn_*	9.4×10^−4^ [*V*_frac_ nmol^−1^ day^−1^]
Bone formation rate	*f_bn_*	5.9098×10^−6^ [*S*_frac_ nmol^−1^ day^−1^]
TGF- 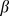 extraction rate	*r_β_*	2.2×10^−10^ [nmol day^−1^]
TGF- 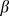 degradation rate	*d_β_*	0.1189 [day^−1^]
Number of cancer cells	*N_c_*	2000
Constant time delay	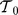	6.02 [day]
Constant time delay	Δ	22.36 [day]
Maximum amount of osteocytes Ocy	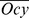	900 [*V*_osteon_^−1^]
Minimum effective amount of TGF-*β* inducing delay	*β*	8×10^−8^ [nmol *V*_frac_^−1^]
Burried osteoblast factor	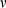	1 [*V*_osteon_ *V*_frac_^−1^]

If the parameter depends on the phenotype 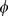, then we give a list of values sorted by increasing order of the phenotype. References and choices for the numerical values of the parameters are discussed in the data analysis section.

Different factors play a role in the prediction of cancer evolution. The presence of characteristic time scales in the development and the dynamics of breast cancer is not so easy to define or to detect. Nevertheless, few considerations can be made. Observing the values of the parameters of the multi scale model, some of which are given in the literature and others are chosen so that their order of magnitude is consistent with the range of values present in similar biological situations (see Sec. Methods), one can a posteriori pinpoint that there are two distinct time scales. The first describes processes occurring at intra-extra cellular time scale and the other, at tissue time scale. Hence, the differences between the two time scales reflect the velocities at which the processes happen at both the cellular level and the tissue level. This implies that fluctuations due to various cells activities, even when they are large, rapidly decay and cancel out. From the tissue scale point of view, we can disregard large rapid cell variations, and consequently, a posteriori, we are justified in adopting the mean-field form of interaction between the cell and the tissue scales.

Looking at various clinical cases and at their respective gene expressions, these two time scales cannot be explicitly determined due to the lack or impossibility of direct experimental measurements. On one hand, using the information for each patient to adjust the model parameters so to better fit each dynamics of the disease, we can observe that there are two distinct time scales, even though they are not unequivocally defined. On the other hand, these two time scales do not identify different steps of the cancer development, nor do they represent two different dynamics. They only express that an abrupt event which suddenly changes the behaviours in a small group of cells may initiate the disease, but its effect will be felt at larger scales only at longer times thanks to the 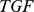-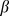 which represents the vector of the interaction.

### The bone Niche Model and the Effect of 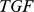-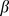 in Bone Remodeling

Breast cancer bone metastases are predominantly osteolytic and accompanied by bone destruction, bone fractures, pain, and hypercalcemia, causing severe morbidity (bone metastases occur in about 70% of patients with advanced breast cancer). Comorbidity addresses the occurrence of different medical conditions or diseases, usually complex and often chronic ones, in the same patient. There is a causal effect, and the bone is a unique microenvironment in which breast cancer thrives [23]. Bone is continuously being formed by osteoblasts and resorbed by osteoclasts, not only to maintain mineral homeostasis but also to cope with the microfractures that occur naturally [24]–[27]. We believe that computational modeling could be very effective in shedding a light across the intrinsic difficulties of integrating evidence obtained from experiments and observations spanning different scales of time and space. There is a growing number of mathematical and computational models investigating the complexity of this dynamics [28]–[34] and the interaction with cancer cells [35], [36]. In the adult skeleton, 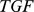-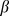 is abundant in the bone matrix, where is released following the initiation of resorption 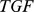-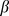 is released from bone matrix [6], [37], [38]. Few recent studies have highlighted the complexity of breast cancer metastasis in bone microenvironment [6], [9], [39], [40]. Although 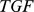-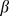 enhances the recruitment and proliferation of osteoblast progenitors, 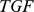-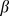 potently inhibits later phases of osteoblast differentiation and maturation and suppresses matrix mineralization [41]–[45]. Osteoblasts are derived from mesenchymal stem cells and their primary function is to synthesize the organic collagenous matrix and orchestrate its mineralization by producing bone matrix proteins including osteocalcin, osteopontin and bone sialoprotein, and providing optimal environmental conditions for crystal formation. Fully differentiated osteoblasts that are surrounded by mineralized bone tissue are called osteocytes and act as mechanosensors in bone tissue. They are the most numerous cells within the bone tissue and scattered evenly through the matrix. With their flattened morphology and long processes, they form a sensory network which allows the detection of abnormal strain situations such as generated by microcracks. Osteocytes are connected to one another and to surface osteoblasts (bone lining cells) via gap junctions. Osteocytes secrete sclerostin which is a master switch to prevent the body from making too much bone. The tuning of sclerostin allows osteocytes to control the osteoblast’s activity in bone formation. Under physiological mechanical stimuli osteocytes prevent osteoclast’s bone resorption by changing the RANKL/osteoprotegerin (OPG) ratio. By communicating these signals to bone lining cells (the second terminally differentiated osteoblast cell type) or secrete factors that recruit osteoclasts, osteocytes initiate the repair of damaged bone. We described a model of the interplay between osteocytes, osteoblasts and osteoclasts. This model focuses on the release of 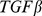 during resorption phases. Pathological conditions can alter the equilibrium between bone resorption and bone formation. Often, disregulations favouring osteoclastogenesis; osteoporosis is an example of negative remodelling: the resorption process prevails on the formation one and this reduces bone density, so increasing the risk of spontaneous fractures. Here we model the release of the 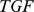-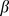 from the bone matrix upon the action of the osteoclasts which favour the breast cancer metastasis and the unbalance of the bone dynamics. While experimental works represent primary sources of parameter values [46], the mathematical and computational recent works, such as [28]–[30], [32], provide a valuable validation and discussion of the range of parameters value. Here we have used parameters values accepted by various literature. There is a growing use of omics genome wide analysis, see [47] among others. In our work we have estimated some parameters from gene expression data. It has been pointed that the difference between an approximate and exact model is usually remarkably smaller than the difference between the exact model and the real biological process [48]. Taking into account the recent experimental results on the central role of osteocytes [24] and the most recent mathematical models, in particular Pivonka [43] we have developed the following conceptual model: first we stress the importance of the osteoblasts forming the lining of cells attached to the bone tissue. It is likely they are in communication with the osteocytes in the bones via a network of canaliculi. When osteocytes die because of fracture there is a loss of communication of the lining osteoblasts with the osteocytes. This happens together with a release of RANKL which is a signal for osteoclasts to arrive and it is a differentiation signal for the osteoblast which may take sometime to happen. We have modeled this delay by using a delay differential equation. The osteoclasts will carry on a resorption of the bone which could be stopped by decrease of RANKL or by the release of 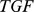-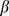 by the bone. Then the osteoblasts with start deploying bone material and proteins and complete the differentiation process by rebuilding the network of canaliculum processes. One can think that cancer cells would take the advantage of absorbing part of the 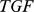-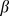 so decreasing the apoptotic probability of the osteoclasts. We have used DDE to implement both the differentiation lapse of the osteoblasts. Delays not only are an explicit representation of the time necessary for spatial transfer of information, but are important also to take into account the presence of other reactions which are not explicitly expressed, or included and the lapse of time for them to happen. The osteoblasts during the bone resorption and bone formation appears in three forms: uncommitted osteoblasts, pre-osteoblasts and active osteoblasts. The differentiation from the first type and the second is accelerated by the presence of 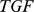-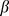 and the differentiation from the second type to the third is inhibited by the 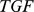-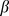. Uncommitted osteoblasts lay on the surface of the bone vessel and their differentiation process is influenced by the osteoclasts’ resorption activity which is mainly triggered by bone fracture (and microfracture). This event propagates and after an average time 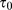 uncommitted osteoblasts begin to differentiate. The presence of 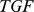-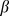 can be of help for their differentiation, or even necessary, but the 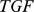-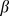 cannot abbreviate it because it does not speed up the travel of information. On the other hand 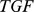-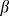 can delay the activation of osteoblasts. The complete and complex differentiation process of the osteoblasts can be summarized from the first stage to the last stage by packing all the complexity of the process inside a delay 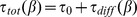 dependent on the 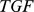-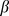, 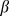. The delay 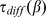 must be positive and finite, because the 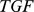-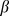 does not preclude the osteoblast activation and second important factor to remark is that the effect of 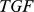-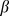 on the osteoblasts is local (at contact) in space and picked (rapid effect and defined delay ) in time. On the other hand the diffusion of 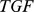-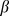, osteoblasts and their concentration are much more sensible to space inhomogeneities and large time distributed events. Our DDE model equations are listed below:
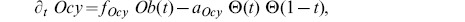
(7)




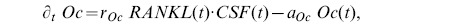
(8)




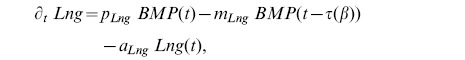
(9)




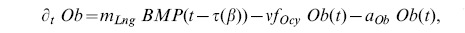
(10)




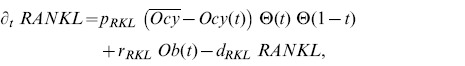
(11)




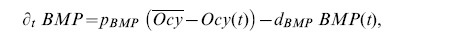
(12)




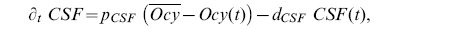
(13)




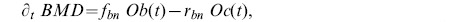
(14)




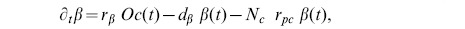
(15)




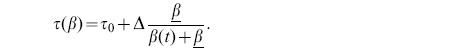
(16)


The equation (7) describes that a microfracture occurring at t = 0, is sensed by osteocytes that undergo apoptosis which ends in about one day [46], [49]. In equation (8) the osteoclasts are recruited to the BMU in response to a combination of RANKL and CSF, and die at a rate 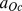; The equation (9) shows that immature osteoblasts are recruited in response to 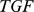-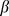 here represented as BMP, and differentiate into mature osteoblasts after 20 days. Mature osteoblasts can either self-bury with a rate 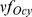 and differentiate into osteocytes at rate 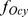 or die at rate 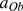, see equation (10). References could be found in [46]. The equation (11) describes that surviving osteocytes secrete RANKL at a rate proportional to the “size of the fracture” i.e. number of osteocytes that underwent apoptosis; osteoblasts produce both RANKL and OPG at rates dependent on their maturity and with characteristic delay [36]. The equations (12, 13) describe that BMP and CSF are secreted by osteocytes in response to the microfracture damage. Both chemicals decay, but CSF is also depleted by osteoclasts; representing the idea that CSF is a chemoattractant and the osteoclasts move on when its concentration becomes low [50], [51]. Note that 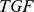-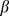 and bone morphogenetic protein (BMP) have often opposite behaviour. In a hair follicle stem cell niche BMP signaling maintains stem cell quiescence while 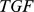-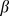 stimulates stem cells both in vivo and in vitro and antagonizes repressive BMP signaling [52].

The equation (14) follows [28] equation where bone is rebuilt at rate 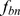 by mature osteoblasts and resorbed at rate 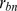 by osteoclasts. The equation (15) describes the 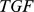-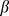 from the resorption action of the osteoclasts.

**Figure 4:**
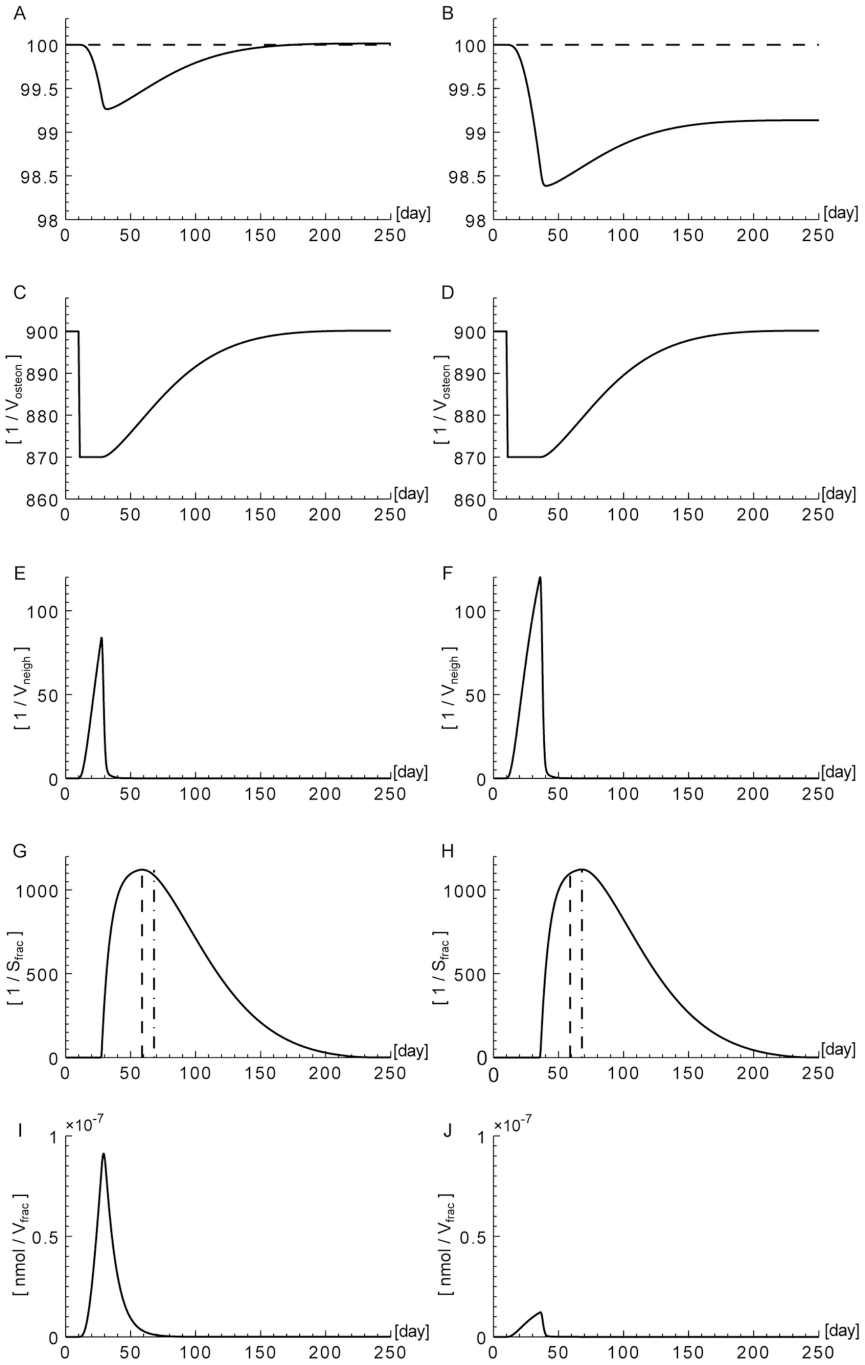
Bone remodelling process. Evolution in time of the bone density (A,B), the osteocytes (C,D), the osteoclasts (E,F), the osteoblasts (G,H), and the 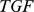-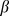 in the bone niche released during the resorption (I,J). Control (first column) versus cancer condition (second column) is shown. The bone densities are expressed in percentage, the osteocytes are in number of cells per osteon volume (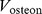), the osteoblasts are in number of cells per fracture surface (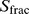), the osteoclast are in number of cells per fracture volume and the 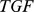-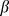 in nanomoles per fracture volume. On the abscissa the time is in days.

In Figure (4) we show the simulated results of the ODE model: Bone density (A, B), number of osteoblasts (C, D), number of osteoclasts (E, F) and 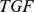-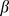 (G, H) compared between control (A, C, E, G) and cancer condition (B, D, F, H). Results show a negative remodelling balance in the cancer case. The values in Figure (4) are expressed in terms of averaged quantities which are the osteon volume, the fracture volume and the fracture surface. Considering a microfracture of 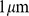 width which caused the damage of a number of osteocytes 30 times smaller than the number of osteocytes in the osteon, the relation between them are 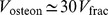 and 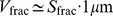, see [53].

The negative balance of the bone density matrix due to the effect of cancer cells in the bone niche, where metastasis occurs, is strongly depending on the number of cancer cells. Even though breast cancer cells find in the bone tissue a richer environment of 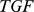-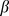 favouring their proliferation, the formation of metastasis is not a very easy and probable event. Different biological defensive systems and causes concur to avoid the formation of metastasis. Nevertheless maybe due to the high number of breast cancer cells detaching from the main tumor and reaching the bone tissue though the vascular system, or maybe due to the occurrence of the rare event in which all the metastatic defensive systems fail, few cancer cells (not necessarily in positions close one another - information neglected in the models proposed) initiate the metastasis. In the very initial development stage, the few metastatic cells do not really affect the bone system. On the other hand, at this stage, a microfracture and the successive remodeling process proceed faster than the increasing of the local cancer cell population. In the early stages of metastasis formation process, we assume that during the remodeling process, the number of the tumoral cells in the bone niche is constant, but after successive microfracture, due to the abundance of 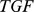-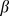, the cancer cell population increases. The more cancer cells increase the more they affect the bone remodeling process. When cancer cells increase in number, on one side, they increase the time of maturation of lining cells into osteoblasts through the action of 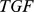-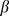 on the BMP, on the other side, they obstruct the tissue re-mineralization. The result is a weaker and less dense bone.

he 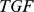-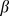 is responsible for the delay in the maturation of osteoblasts. An excessive quantity of 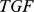-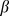 released during the bone remodeling process is the cause of the reduction of the bone density. In the same way, a reduced quantity of 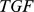-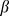 induces a rapid maturation of osteoblasts. This prevents osteoclats resorption in the BMU causing a local increase of bone mass with less structural strength.

Absence of a spatial representation of the bone niche does not allow us to completely describe the dishomogeneities in the bone mineral density caused by the cancer cells in bone tissue which are known as mixed lesions. Cell-to-cell spatial interactions like volume exclusion and chemotaxis are necessary to reproduce mixed lesions. In order to mimic the presence of mixed lesions, we can use our model to simulate the occurrence of multiple fractures in which the intensity of the released 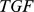-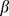 fluctuates randomly. The variability of the bone density accumulated at the end of each remodeling by spatially independent BMUs can be considered as an index for mixed lesions. Hence, high variability will be an indication of mixed lesions, while low variability will represent the cases of osteolytic or osteoblastic lesions depending on negative or positive changes of the averaged bone density, respectively. Nevertheless, such variability is related to the probability distribution used for the intensity of the released 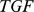-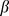.

In Figure (4), we show the evolution of the bone remodeling process after a small fracture when the population of cancer cells in the bone niche is of the order of 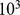. This is sufficient to see that in the local region of bone in which metastasis is developing, the bone density matrix decreases of 1%. The bone mineral density represents a marker to identify the development of metastasis and its effectiveness is based on the causal relation or on comorbidity between breast cancer and osteoporosis.

## Discussion

We have developed a mathematical model describing the multi scalar behaviours of the 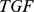-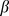, first by representing the actions of the behaviours of the 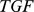-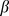 from the intracellular to the extracellular scale using a cellular model which is based on the results of the work published by Laise et al. for epithelial-mesenchymal transformation [14] and which also addresses the important aspect of autocrine/paracrine signaling induced by the protein. Then we have further developed the model to investigate the extracellular mechanisms of action of 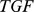-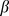 at larger scale and its involvement in cancer. This has led to a tissue model of breast cancer, which usually metastasise to the bone tissue. Therefore, we have also studied the relation between the metastatic cell, the bone tissue and the 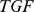-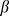. We have identified the 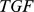-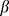 as one key molecule involved in cancer because it is antioncogenic and pro-apoptotic at low concentration and pro-tumorigenic at high concentration due to its mechanobiology properties through the interaction with the cytoskeleton and with the extracellular integrins/E-cad proteins. This multi scale model is based on a multi scale multifunction molecule, in other words it is a sort of hub for apoptosis signaling at one scale and for the cytoskeleton function and cell-to-cell interaction at another scale.

Our work has focused in generating and connecting three models: one cellular model (intracellular and extracellular 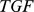-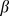) and two tissue models (the breast cancer in the mammary duct and the bone tissue). Each of these models refers to a specific level of organization, and hence it is possible to approximately associate each of them to a specific length scale. Such spatial subdivision of the models is also reinforced by their temporal subdivision in the dynamic processes (cell/tissue scales) and in the cancer disease progress (breast/bone tissues). The differences in temporal scales of the various processes described in these models hamper the aggregation of all these models into a single one. Indeed, the time scales of 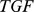-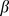 inside the cell are usually much shorter than events at the tissue level. This has been resolved by considering that the variables at the cellular level are passed as “averages” to the tissue level. The information related to the spatial positioning of cells in the tissue has been neglected in order to find results not dependent on the tissue geometry. In other words, we do not consider cellular specialisation in the tissue; therefore, cellular parameters are space and cell type independent, but, if necessary, only phenotype dependent. In this way we avoid an increase in the number of degrees of freedom; furthermore, our approach leads to a model order reduction with no bias for the position of the cell.

The 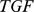-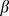 has been reported to show an autocrine control system to inhibit cell replication, thus maintaining the tissue cell homeostasis. We have modeled the process that, following mutations, drives the inhibition into cell cycle progression. So the cell increases the autocriny i.e. the production and endocytosis of 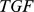-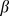 in order to regulate the cell cycle progression, but this results in an increase of tumorigenicity; meanwhile, the 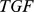-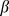 also acts in disrupting the actin cytoskeleton which disrupts the actin and E-cad anchorage; hence, the cell contracts and, as consequence, this increases the activation of extracellular 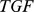-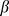; therefore the cell without contact inhibition starts replicating actively. The end of this is a high replication rate of cancer cells that needs to produce 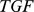-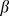 to sustain such growth rate.

In our opinion the downregulation of the 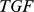-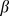 from the internalized 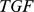-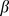 has a defensive effect because it helps to reduce the internalization of 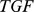-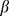 [54]. In the bone niche, bone and cartilage contain large amounts of 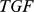-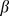 and target cells for 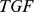-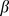 activity. The autocrine and paracrine stimulation by 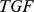-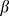 is important for osteoblasts differentiation. The 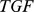-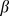 promotes osteoblast differentiation and the 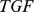-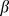 from the osteocytes in the bone make them lining up to the bone. In case of fracture the osteocytes secrete RANKL which activates the osteoclasts. The demolition of the bone around the fracture frees lot of 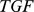-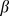 which further attracts the osteoclasts but in the long term induces apoptosis.

The model proposed suggests an ecological perspective of the cancer. Cancer cell changes are associated with alterations in the mechanical properties of the microenvironment; as tumor expands there is an increase in tissue compression and interstitial pressure, generating cell and tissue tension within the confined stroma. These forces induce the release and activation of various growth factors and subsequent changes of the contractility and viscoelasticity of tumor cells. In breast cancer, the different stages of cancer cell determine different mechanical interaction with basement membrane (BM) architecture and ECM. 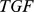-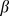 plays a key role in orchestrating the cell-ECM tension. A hyperplasia lesion typically involves the loss of normal cell polarization and organization, the changes in cell-cell contacts and cell-ECM interactions, which result in altered cellular tension and mechano-sensing and transduction. In carcinoma in situ lesions, cell polarity is lost and the lumen is filled by cells. This volume expansion and resistance from the BM and interstitial ECM lead to increased mechanical forces between tumor cells and the stromal matrix. Simultaneously, ECM components are abnormally deposited and remodeled, which results in increased ECM and tissue stiffness, and in turn, cell generated tension. In invasive lesions, tumor cells break down the BM and invade into the interstitial ECM. The reciprocal forces between tumor cells and the ECM continuously increase. The abnormal deposition and remodeling of ECM collagen further increase ECM and tissue stiffness. Tumor cells generate greater tension in response to this increased mechanical stimulation. As tumor cells invade through the BM and ECM, they experience a range of different forces from the dense ECM network.

Although 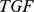-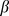 is a growth inhibitor for most epithelial cells, it has multiple and often opposing effects depending on the tissue and the type of cells. Why 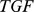-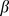 is not regulated more tightly? We believe that cancer is a disease related to the ageing; it is still very rare in young and mature organisms, while is very common in the elderly; so there is no great selection feedback and cancer is a mean to send in apoptosis an aged organism when most of the cells have accumulated mutations, so in some analogy it has the same role that 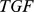-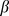 has with single cells when energy becomes limited.

## Methods

We have considered parameter estimates from experiments reported in literature and from published mathematical and computational models. Furthermore, some parameters are explicitly obtained from gene expression analysis, while some other are derived from the models so that their estimation as well as the models’ outputs remain inside ranges of validity consistent with the biological phenomena. The values of the parameters and the boundary conditions for the ODEs and DDEs used in the numerical simulations of the models are given in Tables (1, 2).

In the cellular model, the values for the amount of the various isoforms of 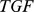-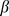 and receptors are extracted from gene expression data. In the specific case shown in Figure (3) and Table (2), where the cellular model and the tissue model are coupled together, we have considered the TGFB2 and TGFR3 isoforms for the cytokine and its receptor respectively.

The 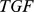-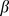 paracrine rate is derived as a direct consequence of the first Fick’s law with an effective diffusion coefficient that takes into consideration the effects of the ECM. The order of magnitude for 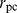 is estimated from the data in [55]–[58] relative to a healthy mammary epithelial cell. The reduction of volume in cancer mammary epithelial cells, as highlighted in [56], is the sign of cell cytoskeletal instabilities due to mutations. As consequence of the cell contractions, a small empty volume, which we have named diffusive region, appears between the cancer cell and the nearest cells of the tissue. The relation between the diffusive region volume and the neighbour volume is 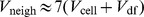 and 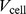 is the averaged volume of a healthy mammary epithelial cell [56]. For the numerical values of 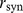 linking the production of 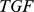-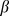 to their gene expression, of the 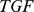-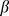 signaling rate to initiate the reactions with the Smad proteins in the cytoplasm and of the 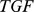-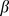 binding rate occurring at the cell surface, we refer to the experimental results in [16] with the assumption that Smad concentrations remain constant. The order of magnitude of mammary epithelial cell proliferation rate is based on the data reported in [59], [60]. For the tissue model, the mutation rate and the probability, 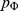, that a mammary cell undergoes malignant mutation showing a strong resistance to 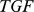-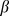 apoptotic signaling, are extracted from [21]. Based on the works [20], [21], we have estimated the proliferation and the apoptosis exponents such that the local cell sub-population with highly malignant phenotype (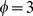) reaches the 10% of the local collective cell population approximatively after 2 years and the cell with phenotype 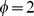 represents those cells with sufficient driver mutations so that the signaling induced by the 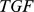-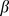 begin to change from anti-tumorigenic to pro-tumorigenic. Most of the parameters used in the bone niche model are taken from the following works: [47], [53], [61]. For the time immature osteoblasts take to differentiate into mature osteoblasts, the rate of osteocytes formation, the apoptosis rate of the osteoblasts and the rate at which the RANKL is released by the osteoblasts, we refer to the results in [46], [62]. For the quantity of 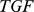-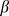 stored in the bone, we refer to the work of Janssens [63].

### Gene Expression Data

Despite the wealth of molecular data (such as sequence and gene expression data) and physiological and pathological data from different populations, there is a lack of cell abundance estimate in the different tissues. Therefore we extracted information from gene expression data. Gene expression patterns supply insight into complex biological networks. Gene expression profiling of the tumor microenvironment during breast cancer progression distinguishes breast carcinomas from normal breast tissues. We have re-analysed several gene expression data related to breast cancer dynamics from the Gene Expression Omnibus (http://www.ncbi.nlm.nih.gov/geo/); after an exploratory analysis and literature analysis, we focused on the following datasets: (accession numbers): GSE14548, GSE33450 and GSE8977. These datasets originate from experimental design on early stages breast cancer progression and tumor microenvironment. Normalization procedures and statistical analysis are performed by using Bioconductor R packages [64]; the background correction and normalization is performed by using PLIER algorithm. PLIER algorithm produces an improved gene expression value [65] as compared to the other algorithms. It accomplishes this by incorporating experimental observations of feature behaviour Specifically, it uses a probe affinity parameter, which represents the strength of a signal produced at a specific concentration for a given probe. The probe affinities are calculated using data across arrays. The Bioconductor package limma was also used to calculate average expression levels, log fold changes and adjusted p-values for each probe. Standard anova and Box plots representation were used to analyse and check out visually the expression levels of these genes for different conditions. We have used gene expression averaged quantities to better unveil the functions of the 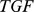-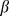 in the cancer dynamics; nevertheless, the model proposed should be used with a pinch of salt. Single patient gene expression values may not be sufficient to catch the specificity of the evolution of the disease for each patient and the role of the 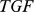-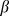. Comorbidities, patients’ previous medical conditions and the exact initial time of breast cancer formation may be unknown, but they affect the prediction of cancer evolution. We have also considered Transforming Growth Factor beta 3 (TGFB3) involved in cell differentiation and which interacts with 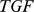-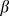 receptor 2 (TGFBR2), a tumor suppressor gene.

### Sensitivity Analysis

The correctness of the proposed multi scale model and its predictive ability on development and progress of the disease strongly depend on the knowledge of the parameters and the respective errors; hence, it is important to rightly identify and estimate the parameters whose the multi scale model is more sensitive. Using the Local Sensitivity Analysis method (LSA) in [66], we compute the first order sensitivity indexes for each of the three models separately. Even though the non-linearity of the models suggests that the second order sensitivity indexes analysis could be relevant to identify the most sensitive model’s parameters couples, for sake of simplicity, we neglect the second order variance decomposition, and we show the LSA for all single parameters. The first order sensitivity index 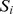 for a parameter 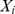 given the parameters 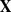 and the output 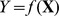 is a pure number defined as 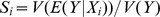, where 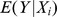 is the conditional expectation value obtained by sampling all the parameters 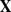 from their prior distributions except 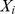 which is maintained constant, 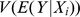 is the variance of 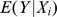 due to the variation of 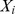 and 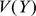 is the variance due to variation of all the parameters 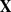.

**Figure 5:**
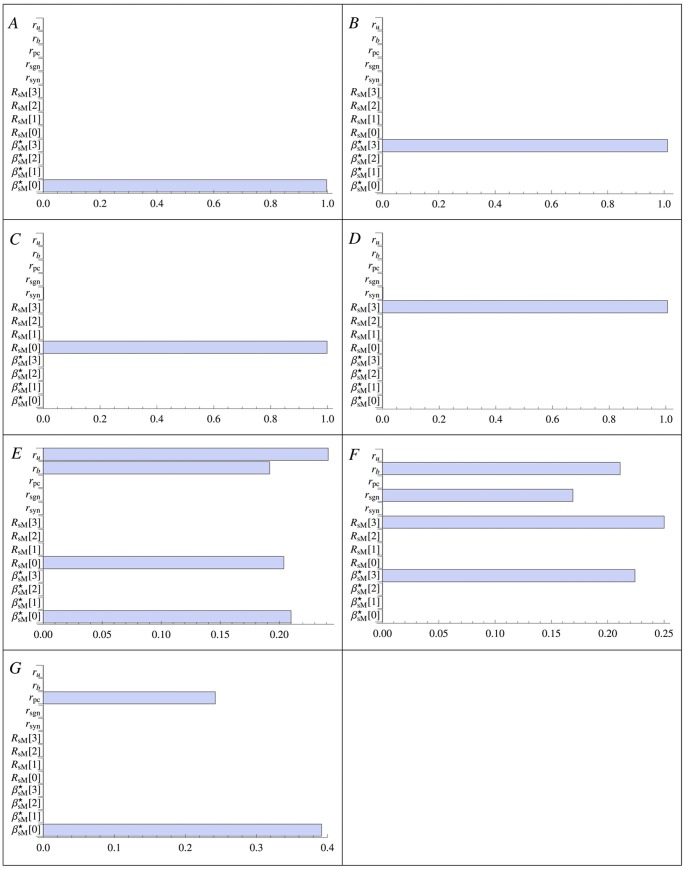
Cellular model LSA. The first order sensitivity index of the cellular model’s parameters for (A,B) the 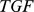-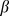, (C,D) the receptors, (E,F) the 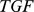-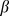 internalized and (G) active 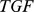-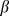 in the diffusive region. On the left column the phenotype 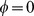 and on the right column 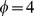 with exception of the 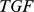-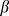 in the diffusive region (G) which is independent of the phenotype.

In the cellular model, the concentration of active 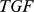-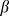 present in the extracellular region is strongly sensitive only to those parameters directly related to their gene expressions, Figure (5.A, 5.B). The same holds true for the 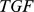-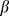 receptors on the cell membrane, Figure (5.C, 5.D). Consequently, accurate information regarding the gene expression are much more important than other parameters to finely tune the outputs of the model and reduce the error on the capability of the model to be predictive. The fact that gene expressions play a fundamental role on the model is because they appear as terms of source of the proteins. Indeed, the other parameter that partially influence the model is the rate of synthesis, which also appears as factor in the source terms of equations (2, 3). Nevertheless, being the prior of the parameters uniformly distributed with a variance of 10% of their values expressed in Table (2), the sensitivity of the system to 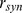 is approximatively two orders of magnitude less than to the gene expressions. On the other hand, the internalized compound not only is sensible to the gene expressions of the 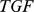-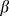 and its receptors, but also to the possibility cells have to internalize the active form of 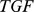-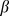 through the receptors. In Figure (5.E, 5.F), we see that the quantity of 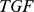-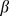 accumulated in the cell cytoplasm depends on the capability of each specific cellular sub-population to respond to the 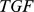-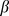 signaling as well as on the capability of healthy cells to ubiquitinate the 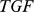-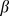. The 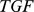-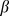 in the diffusive region depends on how fast it diffuses in the surrounding space and on its production by the healthy cells, Figure (5.G). Indeed, we are observing the system, which is in an out-of-equilibrium condition, at a time when the quantity of healthy cells is the predominant cell sub-population component of the total cell population; therefore, in this case, there are no contradictions in saying that the major sources of 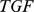-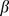 are the healthy cells.

**Figure 6:**
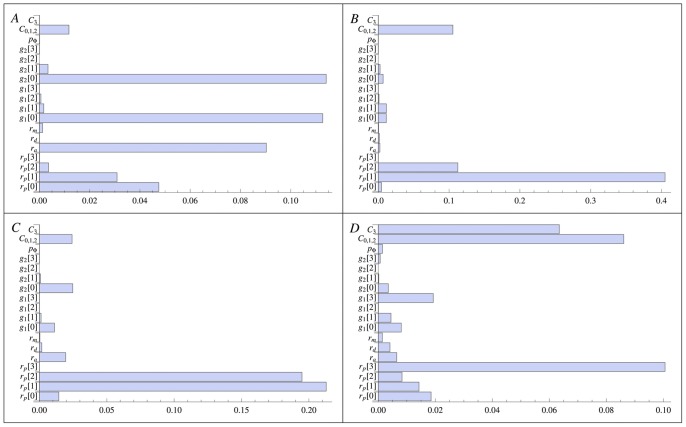
Tissue model LSA. The first order sensitivity index of the tissue model’s parameters for the tissue sub-population: (A) 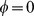, (B) 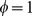, (C) 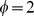, and (D) 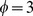.

The sensitivity analysis for the tissue model presents a more complex scenario of the relevant parameters affecting the densities of each cell sub-population based on the cells phenotypes, see Figure (6). Due to the interaction driven by the paracrine exchange of 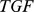-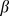, the volume exclusion constraints between the cellular capacities and the strong non-linearity produced by the different cell capabilities of sensing/responding to the 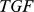-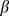, we see that the dynamical evolution of a cell sub-population with specific phenotype depends on parameters strictly non-related to that specific sub-population phenotype. The simplest case is given by the healthy cell sub-population which is prevalently sensitive to its proliferation/apoptosis rates, its exponential indexes, and the healthy cell capacity, Figure (6.A). Depending on the time at which the system is observed, we can see a more or less dependency of the healthy system on the proliferation rate of the pre-tumoral cells. The cell sub-populations characterized by driver mutations show all the same behaviours of being sensitive to the proliferation/apoptosis rates and exponential indexes not only of their same phenotype, but also to those with less aggressive phenotypes, Figure (6.B, 6.C, 6.D). The small dependency on the parameters with phenotype 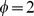 is due to the particular transition role of this cell sub-population in respect to its response/sensing of the 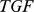-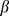 and consequently to the small range of the proliferation/apoptosis indexes (meaning small variance of the prior distribution adopted in the LSA). All cell sub-populations do not show any sensitivity on the capacity 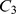, exception done for the highly aggressive tumoral cells which depend on both values of the cell capacities, Figure (6.D).

From the LSA, Figure (6), we see the emersion of an explicit pattern on the parameters which reflects the behavioural evolution of the tissue in terms of dynamics. In fact, even though all the cells compete for limited room by obstructing or killing the cells with different phenotypes, the number of tumoral cells and their survival, during the early stage of cancer, are strongly linked to their phenotypical ancestors. Hence, the two major sub-systems of the tissue, healthy tissue and tumor, show the dual behaviour of two resources competing systems like in the predator-prey model, on one side and of two shearing 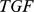-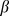 systems in which one try to stabilize via 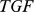-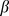 the other sub-system without any success because the driver mutations in the aggressive cancer cells have changed the apoptotic signaling of the 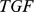-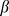 into a tumor promoter, on the other side.

**Figure 7:**
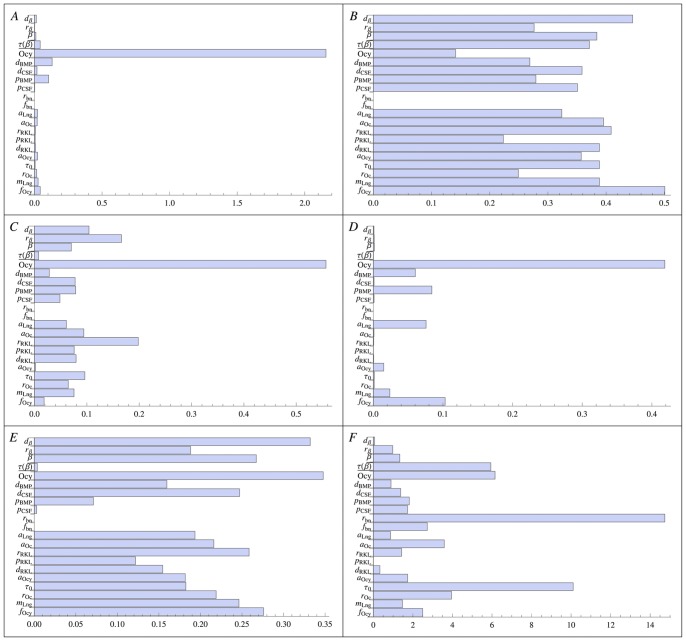
Bone model LSA. The first order sensitivity indexes of the bone model’s parameters for (A) osteocytes, (B) osteoclasts, (C) lining cells, (D) osteoblasts, (E) RANKL, and (F) bone mineral density.

The LSA in the bone model shows two groups of variables: those which are mostly sensitive to the number of osteocytes in the BMU before a fracture occurs, but do not show any relevant sensitivity to the 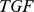-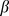 related parameters, and those variables which, even though depend on the osteocytes, are sensitive to the delay induced by the 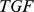-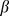 as well as to the other parameters responsible to the high concentration of 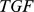-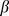 in the bone niche, Figure (7). The number of lining cells depends on the time they take to differentiate and hence they are sensitive to the 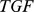-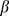, Figure (7.C). On the other hand, mature osteoblasts are not very sensitive to the 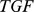-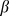, but are sensitive to the apoptotic rate of the lining cells because soon or later all the lining cell, which do not undergo apoptosis, follow through maturation, Figure (7.D). We have also observed that the RANKL presents a strong sensitivity to the 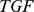-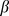 which is an indication that the small quantity of RANKL released by the osteoblast compared to that released by the damaged osteocytes network is important to affect the dynamics of the bone remodelling, Figure (7.E). Consequently, the dependence of osteoclasts on 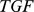-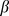 is due to the RANKL, Figure (7.B). The mineral bone density is the variable with the highest sensitivity to the 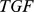-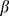, Figure (7.F), showing that the resorption and remineralization are carefully synchronized by the 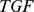-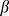 which regulates the time interval between osteoclasts and osteoblasts actions.

It is important to highlight that the roles of osteoclasts and breast cancer cells, in the bone niche, are the same in terms of 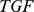-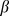, but are extremely different in their resultant actions. The first (osteoclasts) produce 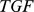-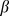 to induce a delay necessary to complete the bone resorption and to carefully balance the maturation of lining cells, but at the same time, they are miners releasing a strong chemoattractant for the tumoral cells from the breast lobular duct. The last (breast cancer cells) remaining in the bone niche because rich of 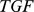-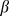, which is a fundamental resource for their survival, release 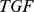-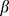 to prolongates the mining of the osteoclasts, to make themselves space and to maintain their cell cycle progression.

As previously stated the LSA is a useful tool to determine the importance of the parameters based on the fact that a parameter of the model with high sensitivity and low variance should be carefully chosen and/or measured because highly affecting the outcomes of the model while a parameter with low sensitivity and high variance does not influence the system dynamics. Furthermore, we have used the LSA to reveal the relationships of and the patterns of variables depending parameters in the tissue and in the bone model.
